# In Vivo Brain Imaging, Biodistribution, and Radiation Dosimetry Estimation of [^11^C]Celecoxib, a COX-2 PET Ligand, in Nonhuman Primates

**DOI:** 10.3390/molecules23081929

**Published:** 2018-08-02

**Authors:** J. S. Dileep Kumar, Bing Bai, Francesca Zanderigo, Christine DeLorenzo, Jaya Prabhakaran, Ramin V. Parsey, J. John Mann

**Affiliations:** 1 Molecular Imaging and Neuropathology Division, New York State Psychiatric Institute, 1051 Riverside Drive, New York, NY 10032, USA; Francesca.Zanderigo@nyspi.columbia.edu (F.Z.); prabha@nyspi.columbia.edu (J.P.); John.Mann@nyspi.columbia.edu (J.J.M.); 2 Now at Department of Radiology, University of Southern California, Los Angeles, CA 90089, USA; bing.bbai@gmail.com; 3 Department of Psychiatry, Columbia University, New York 10032, NY, USA; 4 Now at Department of Psychiatry, Stony Brook School of Medicine, Stony Brook, New York, NY 11794, USA; Christine.DeLorenzo@stonybrookmedicine.edu (C.D.); Ramin.Parsey@stonybrookmedicine.edu (R.V.P.)

**Keywords:** PET, COX-2, celecoxib, dosimetry, biodistribution, brain

## Abstract

COX-2 selective inhibitors (COXIBs) are non-steroidal anti-inflammatory drugs (NSAIDs), with fewer side effects compared with non-selective NSAIDs, and are used for the treatment of arthritis, headaches, and other inflammatory diseases of the brain and peripheral tissues. Radiolabeled COXIBs may permit positron emission tomography (PET) imaging of COX-2 localization and activity in diseases, enable monitoring of inflammatory processes, and determine target occupancy of COX-2 activity by NSAIDs, thus, accelerating the development of novel CIXIBs. We synthesized [^11^C]celecoxib, one of the COXIBs and a prescription drug, and here report its in vivo uptake in the brain, whole body biodistribution, and radiation dosimetry in baboons using PET. Brain imaging experiments were performed in one baboon and whole body PET scans were performed in triplicates in two male baboons using an ECAT ACCEL (Siemens Medical Solutions, Inc. Knoxville) under anesthetic conditions. PET studies in baboons show that [^11^C]celecoxib penetrates the blood brain barrier (BBB) and accumulates in the brain, followed by a washout of radioactivity. The liver has the highest residence time and the gallbladder is the critical organ for [^11^C]celecoxib. Organ Level Internal Dose Assessment (OLINDA) estimates indicate that the maximum permissible single study dosage of [^11^C]celecoxib in humans is 1110 MBq (30 mCi) for both males and females under the 21 CFR 361.1 dose limit for research subjects.

## 1. Introduction

**Figure 1:**
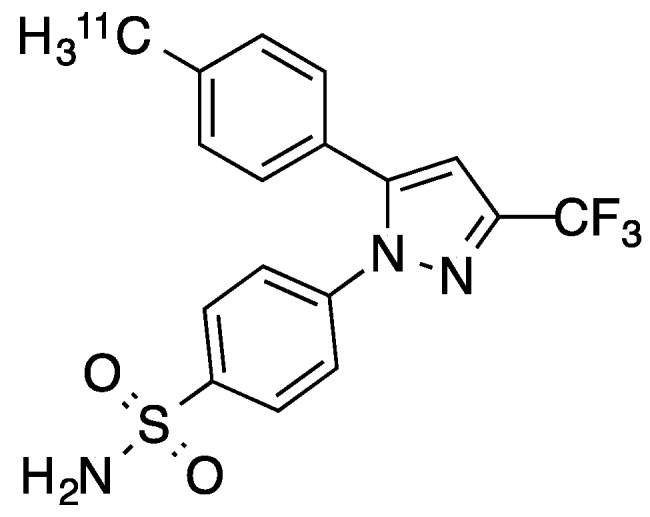
Chemical structure of [^11^C]celecoxib.

Cyclooxygenase (COX) is an enzyme involved in the biosynthesis of prostaglandins, prostanoids, and thromboxins from arachidonic acid and drugs that block COX-1 and COX-2 isoenzymes that are non-steroidal anti-inflammatory drugs (NSAIDs) [1,2,3]. Of these two isoenzymes of COX, COX-1, is constitutively expressed in many tissues and is responsible for the production of prostanoids associated with normal haemostatic functions [4]. In contrast, COX-2 is an inducible enzyme involved in cellular responses associated with inflammation [1,2,3,4]. Another variant enzyme of the COX family, COX-3, has been recently identified and is believed to mediate fever; its inhibition is involved in the antipyretic effect of NSAIDs [5]. Expression of COX-2 proteins and mRNA in human subjects show that COX-2 levels in normal tissues are the highest in the kidney followed by brain, then the spleen, liver, heart, and intestine [6,7,8]. COX-2 expression is upregulated in the inflammatory process in conditions, such as cancers, arthritis, autoimmune disorders, ischemic heart disease, stroke, organ rejection, and neurodegenerative diseases, like Alzheimer’s and Parkinson’s diseases [9,10,11,12,13]. Due to the involvement of COX-2 in diseases or disease processes, quantifying COX-2 expression is a potential biological marker for early diagnosis and for monitoring disease progression. Measurement of target occupancy allows drug development of new COX-2 NSAIDs, and an indicator of effective treatment. Since, COX-2 is constitutively expressed in many organs, and COX-2 inhibitors are known to inhibit platelets in the blood, side effects associated with COX-2 inhibitors as therapeutics are of major concern. The low injected mass of a radioligand that is required for quantitative PET imaging means that highly selective COX-2 NSAID drugs are potential candidates to be radiolabeled with PET isotopes for use as imaging agents. We chose celecoxib, a highly selective COX-2 inhibitor (COX-2 and COX-1 IC_50_ values of celecoxib are 40 and 17,000 nM, respectively), and is safe to use in humans since it is an FDA-approved prescription drug, as a candidate for PET ligand development [14,15,16]. We previously radiolabeled celecoxib with [^18^F]isotope and found that the tracer undergoes rapid [^18^F]defluorination in rodents [17]. However, biodistribution and pharmacokinetics studies with unlabeled or [^14^C]-labeled celecoxib showed that the ligand did not undergo defluorination in rat and human subjects [18,19]. Therefore, the de[^18^F]fluorination of [^18^F]celecoxib is not likely due to the enzymatic process, but possibly related to its auto radiolysis in vivo. Although de[^18^F]fluorination was relatively slower in nonhuman primates compared with rodents [17], we decided to pursue [^11^C]celecoxib synthesis and employed a Stille coupling reaction to develop a one pot synthesis [20]. Herein, we describe the brain uptake, whole body biodistribution, and dosimetry of [^11^C]celecoxib in non-human primates (Figure 1).

## 2. Results and Discussion

### 2.1. Radiochemistry of [^11^C]Celecoxib and Cross Selectivity of Celecoxib to Brain Targets 

[^11^C]Celecoxib was synthesized via a one pot procedure developed based on our previously published method in 40 min at the end of bombardment (EOB) and in 8 ± 2% yield at EOB with >95% radiochemical purity along with a molar activity of 39 ± 6.6 GBq/micromol (n = 12). Celecoxib did not show significant affinity to a variety of competitive brain receptors, transporters, biogenic amines, and proteins (Ki > 10 microM) (Table 1) based on the National Institute of Mental Health–Psychoactive Drug Screening Program (NIMH-PDSP) binding assays [21,22]. 

### 2.2. PET Imaging of [^11^C]Celecoxib in Baboon Brain

**Figure 2:**
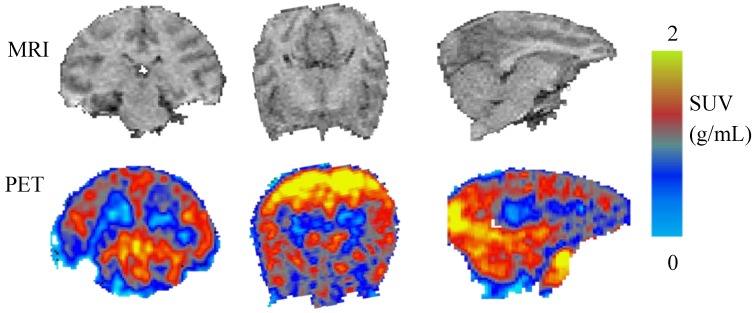
MRI and co-registered positron emission tomography (PET) images (sum of radioactivity between 0 and 120 min after injection) of [^11^C]celecoxib. PET scan in baboon brain.

**Figure 3:**
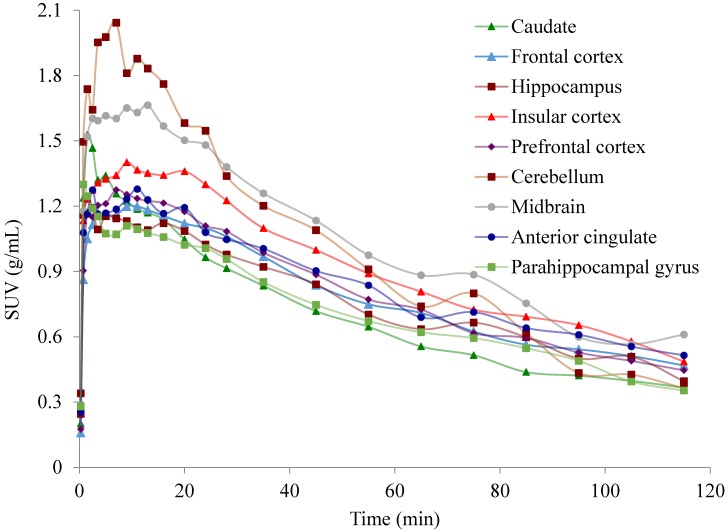
Decay-corrected time activity curves of [^11^C]celecoxib in the baboon brain normalized by injected radioactive dose and body weight.

**Figure 4:**
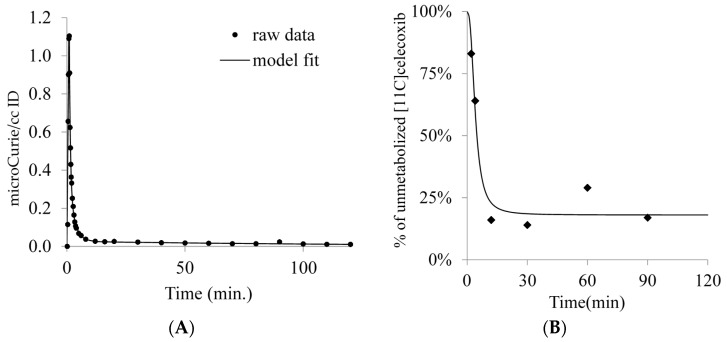
(**A**) Decay-corrected plasma activity of [^11^C]celecoxib in the baboon. (**B**) Unmetabolized parent fraction of [^11^C]celecoxib in baboon plasma.

**Figure 5:**
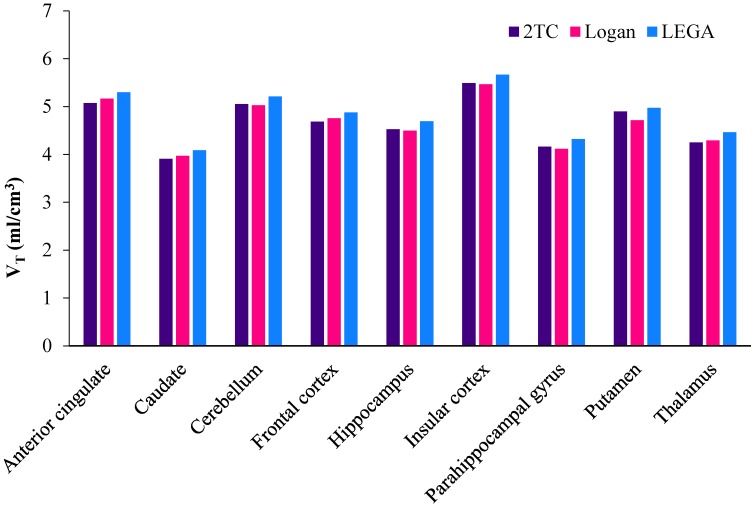
Metabolite-corrected total volume of distribution of [^11^C]celecoxib in the baboon brain.

PET imaging in an anesthetized baboon, corresponding to an injected mass ≤1.5 micrograms shows that [^11^C]celecoxib penetrates the blood brain barrier (BBB) and the radioactivity is retained in the brain (Figure 2). Decay corrected time activity curves (TACs) expressed in the standard uptake value (SUV) indicated a peak uptake of the radiotracer at an interval between three to 10 min post injection in various brain regions, followed by a gradual washout of activity. [^11^C]celecoxib exhibited a somewhat heterogeneous distribution in the baboon brain (Figure 2 and Figure 3). The radioactivity levels in baboon plasma peaked around one min post injection, followed by a gradual clearance (Figure 4A). HPLC analyses of the plasma samples indicated a fast metabolism of [^11^C]celecoxib, with 17% unmetabolized radioligand at 90 min (Figure 4B). Two-tissue compartment (2-TC), Logan plot, and likelihood estimation in graphical analysis (LEGA) yielded comparable estimates of [^11^C]celecoxib total volume of distribution (V_T_) across most regions (Figure 5) [23,24,25]. On average, the highest V_T_ was found in the midbrain, followed by the cortical regions (Figure 5).

### 2.3. Whole Body Biodistribution and Radiation Dosimetry Estimation of [^11^C]Celecoxib in Baboons

**Figure 6:**
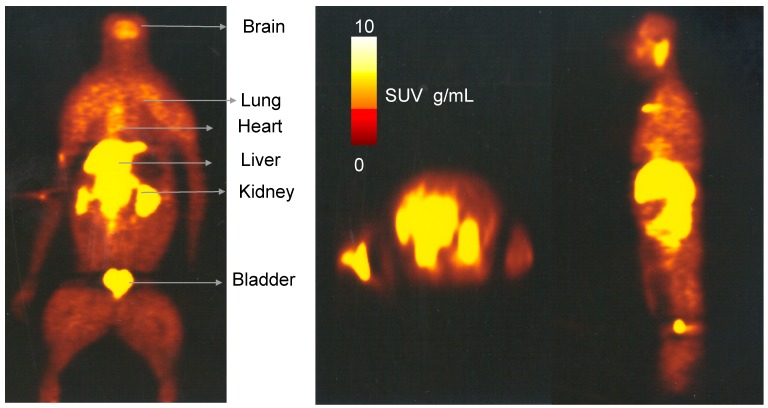
A representative biodistribution and sum of slices over 110 min whole body image of [^11^C]celecoxib in the baboon (Left: Transaxial; middle: Coronal; right: Sagittal).

**Figure 7:**
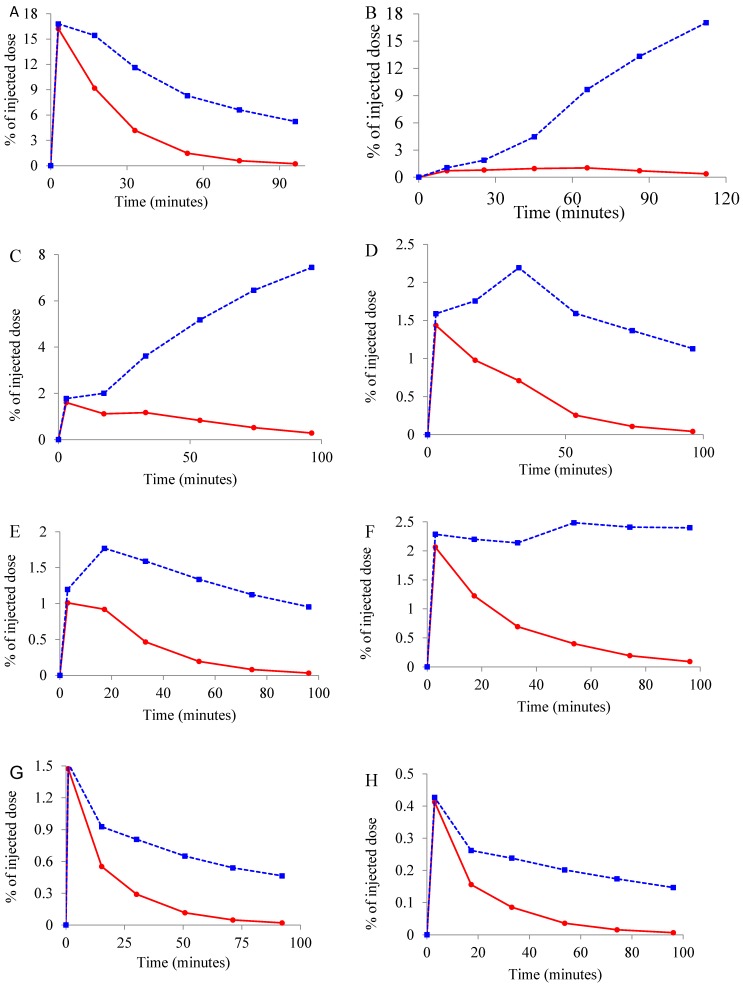
Time activity curves (TACs) of [^11^C]celecoxib in different organs of the baboon. Data are the averages determined from two adult male baboons in six occasions (The blue curves (dotted lines) are decay-corrected and the red curves (solid lines) are non-decay-corrected TACs for each organ) (**A**: Liver; **B**: Urinary bladder; **C**: Gallbladder; **D**: Kidney, **E**: Head; **F**: Intestine; **G**: Heart; **H**: Lung).

All animals tolerated the intravenous injection of [^11^C]celecoxib. There was no significant physiological effect, such as diastolic blood pressure, heart rate, respiratory rate, or rectal temperature, with the administration of [^11^C]celecoxib in baboons. Figure 6 shows the representative whole body distribution of [^11^C]celecoxib. Radioligand exhibited binding to the brain, heart, and kidney, the organs known for the constitutive expression of COX-2. The liver, gallbladder, and urinary bladder showed the highest retention of radioactivity. Decay-corrected and non-decay-corrected time activity curves for various organs are reported in Figure 7. The area under the curve was obtained from the sum of trapezoidal integration and the integral of the exponential functions from time zero to infinity. The liver shows the highest residence time (Table 2). Residence times were used as an input function for dosimetry estimations with the Organ Level Internal Dose Assessment (OLINDA) program.

Table 3 and Table 4 show the dosimetry estimates extrapolated for adult male (70 kg) and adult female (57 kg) human subjects, respectively. This data indicate elimination of [^11^C]celecoxib via the hepatobiliary and renal systems. The gallbladder received the highest estimated radiation dose for males and females and it was the critical organ (1.48E-02 rad/mCi and 1.65E-02 rad/mCi), respectively.

## 3. Experimental

### 3.1. Brain PET Imaging and Quantification of [^11^C]Celecoxib in Baboons 

All animal experiments were carried out with the approval of the Institutional Animal Care and Use Committees (IACUC) of Columbia University Medical Center and the New York State Psychiatric Institute (IACUC approval no.# AC-AAAA0869). Brain PET scanning was performed in an anesthetized male baboon with an ECAT EXACT HR+ scanner (CPS/Knoxville, TN, USA) as described previously [26]. An arterial line was placed to collect blood samples for determination of the arterial input function. The head was positioned at the center of the field of view, and a 10 min transmission scan was performed before the tracer injection. [^11^C]celecoxib was injected (114.7 MBq) as intravenous bolus over 30 s, and emission data were collected for 120 min in 3-dimensional mode. Plasma samples were collected using an automated system every 10 s for the first 2 min, and manually thereafter for a total of 34 samples (0.75 mL) up to 120 min post injection. All PET images were co-registered with magnetic resonance images (MRI) using FMRIB’s Linear Image Registration Tool (FLIRT). Regions of interest (ROIs) drawn on the animal’s MRI scan were transferred to co-registered automated image registration (AIR) frames of PET data. Total plasma counts and high-performance liquid chromatography (HPLC) assay of six of the collected arterial blood samples (~2 mL) provided amounts of total radiotracer counts and the unmetabolized parent compound in plasma [26]. The metabolite-corrected arterial input function was then obtained by multiplying the parent fraction curve (fitted using a Hill function [27]) by the individual total plasma counts, and by fitting the resulting data points with a combination of a straight line (before the peak) and the sum of three decreasing exponentials (after the peak). Regional total distribution volumes (V_T_, ml of plasma/cm^3^ of tissue) of [^11^C]celecoxib were estimated using the 2-TC and graphical analyses with Logan and LEGA and the metabolite-corrected arterial input function [23,24,25]. 

### 3.2. Whole Body Biodistribution and Radiation Dosimetry Estimations of [^11^C]Celecoxib in Baboons

Whole body biodistribution and dosimetry estimations of [^11^C]celecoxib in baboons were performed based on a protocol we developed earlier [28,29,30,31,32]. In brief, whole body PET scans were performed in 2 adult male baboons on 6 occasions with an ECAT ACCEL (Siemens Medical Solutions, Inc., Knoxville, TN, USA) with an intravenous injection of 111 ± 18.5 MBq [^11^C]celecoxib in two-dimensional mode for 120 min in 5 bed positions. Image analyses were performed with software Medx 3.3 (Sensor Systems, Sterling, VA, USA). Organs that were clearly visible on all scans were identified for ROIs and dosimetry analyses. The area under the curve was obtained by trapezoidal integration of the radioligand time activity curves. The organ residence times were calculated from the ratio of area under the TACs to infinity over the initial total body activity. The Organ Level Internal Dose Assessment (OLINDA) software (Hermes Medical Solutions Inc., Greenville, NC, USA) was used to calculate the absorbed radiation dose [33]. The residence times were calculated based on the adult male (70 kg) and adult female (57 kg) human phantoms in OLINDA. The activity not included in the organ ROIs, or the remainder blood activity, was not measured. In the residence time calculation, an organ called the remainder of the body was obtained from the sum of all organ activity subtracted from the total dose.

Images were transferred into the image analysis software Medx 3.3 (Sensor Systems, Sterling, VA, USA). ROIs were drawn around organs that were identified clearly on all scans. The area under the curve was obtained by trapezoidal integration of the radioligand time activity curves (TACs) data points. The organ residence times were then calculated from the ratio of area under the TACs to infinity over the initial total body activity. The absorbed radiation dose was calculated using the Organ Level Internal Dose Assessment (OLINDA) software [33]. The residence times were extrapolated to the adult male (70 kg) and adult female (57 kg) human phantoms in OLINDA. The remainder of the blood activity (activity not included in the organ ROIs) was not measured. In the dosimetry calculation, an “organ” called the rest of the body was used and its TAC was obtained from the sum of all organ activity subtracted from the total dose and entered into the OLINDA program as the “remainder of the body”.

## 4. Conclusions

All animals tolerated intravenous doses of [^11^C]celecoxib in baboons, with no meaningful physiological or pharmacological effects. [^11^C]celecoxib penetrated the BBB and exhibited a somewhat heterogeneous binding in the baboon brain with washout kinetics amenable for tracer kinetic modeling. Biodistribution and dosimetry estimation in baboons suggest that the single study dose limit of [^11^C]celecoxib, extrapolated to male and female human subjects, is 30 mCi, which is below the dose limit for research subjects based on the 21 CFR 361.1. This dose limit allows two or three PET sessions up to 10 mCi single doses of [^11^C]celecoxib in a human subject per year. The gallbladder exhibits the critical organ for [^11^C]celecoxib for male and female subjects. Therefore, existing human safety data and the dosimetry findings make [^11^C]celecoxib suitable for human use for proof of concept studies. 

## Figures and Tables

**Table 1:** Affinity and selectivity of celecoxib.

Targets	Affinity (nM)	Targets	Affinity (nM)
COX-2^14^	40 nM	COX-1^14^	17,000
5-hydroxytryptamin 1-7 receptors	>10,000	Adenosine receptors	>10,000
Alpha1,2 receptors	>10,000	AMPA receptors	>10,000
Beta1-3 receptors	>10,000	Calcium channel receptor	>10,000
Benzodiazepine receptors	>10,000	Dopamine 1-5 receptors	>10,000
Cannabinoid 1,2 receptors	>10,000	Dopamine transporter	2487
Delta opioid receptor	>10,000	Prostanoid receptors	>10,000
Prostaglandin receptors	>10,000	Histamine 1-4 receptors	>10,000
Gamma-aminobutyric acid receptors	>10,000	Kainate receptors	>10,000
Imidazoline receptor	>10,000	Kappa Opioid receptor	>10,000
Human Ether-à-go-go	>10,000	Muscarinic 1-5 receptors	>10,000
Norepinephrine transporter	>10,000	mGluR receptors	>10,000
Neurokinin receptors	>10,000	Mu opioid receptor	>10,000
Neurotensin receptors	>10,000	Serotonin transporter	>10,000
Sodium Channel	>10,000	Smoothened receptor	>10,000
Nociceptin opioid peptide receptor	>10,000	Sigma 1, 2 receptors	>10,000
NMDA receptors	>10,000	VMAT receptors	>10,000
Vanilloid receptor	>10,000	NR2B receptor	>10,000
Oxytocin receptors	>10,000	Protein kinase C receptors	>10,000
Peripheral benzodiazepine receptor	>10,000	Vasopressin receptors	>10,000

**Table 2:** Average residence time of [^11^C]celecoxib (unit: Bq·h/Bq).

Organ	Residence Time
Gall Bladder	0.015627
Urinary Bladder	0.01683
Brain	0.006497
Heart	0.00464
Small Intestine	0.010684
Kidneys	0.015125
Liver	0.063269
Lungs	0.002582
Spleen	0.001115
Stomach wall	0.003536
Vertebra	0.000769
Remainder	0.349441

**Table 3:** Dosimetry estimation of [^11^C]celecoxib extrapolated to adult males.

Organ	mGy/MBq	Mrad/mCi	Mrad/ID	% Limit
Adrenals	3.45 × 10^−3^	1.28 × 10^1^	3.83 × 10^2^	7.7
Brain	1.82 × 10^−3^	6.73	2.02 × 10^2^	4.0
Breasts	2.04 × 10^−3^	7.55	2.26 × 10^2^	4.5
**Gallbladder Wall**	3.99 × 10^−2^	**1.48** × 10^2^	**4.43** × 10^3^	**88.6**
LLI Wall	2.95 × 10^−3^	1.09 × 10^1^	3.27 × 10^2^	6.5
Small Intestine	6.17 × 10^−3^	2.28 × 10^1^	6.85 × 10^2^	13.7
Stomach Wall	4.66 × 10^−3^	1.72 × 10^1^	5.17 × 10^2^	10.3
ULI Wall	3.55 × 10^−3^	1.31 × 10^1^	3.94 × 10^2^	7.9
Heart Wall	5.34 × 10^−3^	1.98 × 10^1^	5.93 × 10^2^	11.9
Kidneys	1.51 × 10^−2^	5.59 × 10^1^	1.68 × 10^3^	33.5
Liver	1.18 × 10^−2^	4.37 × 10^1^	1.31 × 10^3^	26.2
Lungs	1.97 × 10^−3^	7.29	2.19 × 10^2^	4.4
Muscle	2.43 × 10^−3^	8.99	2.70 × 10^2^	5.4
Ovaries				
Pancreas	3.65 × 10^−3^	1.35 × 10^1^	4.05 × 10^2^	8.1
Red marrow	2.23 × 10^−3^	8.25	2.48 × 10^2^	8.3
Osteogenic Cells	3.28 × 10^−3^	1.21 × 10^1^	3.64 × 10^2^	7.3
Skin	1.90 × 10^−3^	7.03	2.11 × 10^2^	4.2
Spleen	3.16 × 10^−3^	1.17 × 10^1^	3.51 × 10^2^	7.0
Testes	2.34 × 10^−3^	8.66	2.60 × 10^2^	8.7
Thymus	2.40 × 10^−3^	8.88	2.66 × 10^2^	5.3
Thyroid	2.25 × 10^−3^	8.33	2.50 × 10^2^	5.0
Urinary Bladder Wall	1.38 × 10^−2^	5.11 × 10^1^	1.53 × 10^3^	30.6
Uterus				
Total Body	2.81 × 10^−3^	1.04 × 10^1^	3.12 × 10^2^	
	**mSv/MBq**	**mrem/mCi**	**mrem/ID**	
Effective dose equivalent	6.97 × 10^−3^	2.58 × 10^1^	7.74 × 10^2^	
Effective dose	4.04 × 10^−3^	1.49 × 10^1^	4.48 × 10^2^	14.9

**Table 4:** Dosimetry estimation of [^11^C]celecoxib extrapolated to adult females.

Organ	mGy/MBq	Mrad/mCi	Mrad/ID	% Limit
Adrenals	4.37 × 10^−3^	1.62 × 10^1^	4.85 × 10^2^	9.7
Brain	2.18 × 10^−3^	8.07	2.42 × 10^2^	4.8
Breast	2.60 × 10^−3^	9.62	2.89 × 10^2^	5.8
**Gallbladder Wall**	4.47 × 10^−2^	**1.65** × 10^2^	**4.96** × 10^3^	**99.2**
LLI Wall	3.73 × 10^−3^	1.38 × 10^1^	4.14 × 10^2^	8.3
Small Intestine	7.22 × 10^−3^	2.67 × 10^1^	8.01 × 10^2^	16.0
Stomach Wall	5.65 × 10^−3^	2.09 × 10^1^	6.27 × 10^2^	12.5
ULI Wall	4.43 × 10^−3^	1.64 × 10^1^	4.92 × 10^2^	9.8
Heart Wall	6.94 × 10^−3^	2.57 × 10^1^	7.70 × 10^2^	15.4
Kidneys	1.66 × 10^−2^	6.14 × 10^1^	1.84 × 10^3^	36.9
Liver	1.56 × 10^−2^	5.77 × 10^1^	1.73 × 10^3^	34.6
Lungs	2.54 × 10^−3^	9.40	2.82 × 10^2^	5.6
Muscle	3.03 × 10^−3^	1.12 × 10^1^	3.36 × 10^2^	6.7
Ovaries	3.90 × 10^−3^	1.44 × 10^1^	4.33 × 10^2^	14.4
Pancreas	4.53 × 10^−3^	1.68 × 10^1^	5.03 × 10^2^	10.1
Red marrow	2.72 × 10^−3^	1.01 × 10^1^	3.02 × 10^2^	10.1
Osteogenic Cells	4.39 × 10^−3^	1.62 × 10^1^	4.87 × 10^2^	9.7
Skin	2.38 × 10^−3^	8.81	2.64 × 10^2^	5.3
Spleen	3.90 × 10^−3^	1.44 × 10^1^	4.33 × 10^2^	8.7
Testes				
Thymus	3.09 × 10^−3^	9.92	2.97 × 10^2^	5.9
Thyroid	2.68 × 10^−3^	6.77 × 10^1^	2.03 × 10^3^	40.6
Urinary Bladder Wall	1.83 × 10^−2^	1.53 × 10^1^	4.60 × 10^2^	9.2
Uterus	4.14 × 10^−3^	0.00 × 10	0.00	0.0
Total Body	3.53 × 10^−3^	1.31 × 10^1^	3.92 × 10^2^	
	**mSv/MBq**	**mrem/mCi**	**mrem/ID**	
Effective dose equivalent	8.35 × 10^−3^	3.09 × 10^1^	9.27 × 10^2^	
Effective dose	4.83 × 10^−3^	1.79 × 10^1^	5.36 × 10^2^	17.9
